# Pharmacogenetics of anticancer monoclonal antibodies

**DOI:** 10.20517/cdr.2018.20

**Published:** 2019-03-19

**Authors:** Dmitrii Shek, Scott A. Read, Golo Ahlenstiel, Irina Piatkov

**Affiliations:** ^1^Blacktown Clinical School, Western Sydney University, Blacktown, NSW 2148, Australia.; ^2^Storr Liver Centre, The Westmead Institute for Medical Research, The University of Sydney, Westmead, NSW 2145, Australia.; ^3^Blacktown Hospital, Blacktown, NSW 2148, Australia.

**Keywords:** Pharmacogenetics, pharmacogenomics, immune-checkpoint proteins, monoclonal antibodies, cancer immunotherapy, personalized medicine

## Abstract

Pharmacogenetics is the study of therapeutic and adverse responses to drugs based on an individual’s genetic background. Monoclonal antibodies (mAbs) are a rapidly evolving field in cancer therapy, however a number of newly developed and highly effective mAbs (e.g., anti-CTLA-4 and anti-PD-1) possess pharmacogenomic profiles that remain largely undefined. Since the first chemotherapeutic mAb Rituximab was approved in 1997 by the US Food and Drug Administration for cancer treatment, a broad number of other mAbs have been successfully developed and implemented into oncological practice. Nowadays, mAbs are considered as one of the most promising new approaches for cancer treatment. The efficacy of mAb treatment can however be significantly affected by genetic background, where genes responsible for antibody presentation and metabolism, for example, can seriously affect patient outcome. This review will focus on current anticancer mAb treatments, patient genetics that shape their efficacy, and the molecular pathways that bridge the two.

## Introduction

### The evolution of monoclonal antibodies in cancer treatment

Over the last 20 years, immunotherapy has become established as one of the most promising and effective therapeutic strategies targeting cancer. Monoclonal antibodies (mAbs) in particular have revolutionized the treatment of hematologic and solid malignancies^[1]^. In 1997, the first chemotherapeutic mAb (Rituximab) was approved by the FDA for clinical use^[2]^, and was quickly followed by numerous other mAbs for a range of malignancies [Table 1]. The generation of therapeutic mAbs began over 20 years previous, where the first mAbs were synthesized using hybridoma technologies from murine sources (-omab). The method consisted of mouse immunisation against a specific antigenic epitope, followed by extraction of splenic B-lymphocytes and their fusion with immortal myeloma cells. The resulting cell clones produced antibodies towards a single epitope, hence the name monoclonal (single clone) antibodies. Unfortunately, the use of such mAbs was restricted due to the development of an immune response against the mouse derived antibodies, termed HAMA (human anti-mouse antibody response)^[3]^. The development of mAbs has since evolved quickly, resulting in subsequent generations of mAbs that were chimeric (-ximab), humanised (-zumab) and fully human (-umab). Chimeric mAbs are composed of variable regions derived from mice, and the remainder [constant domains of heavy chain - C_H (1-3)_] from human or other animals. Humanised mAbs are engineered from human sources and contain only a mouse derived antigen-binding fragment representing ~5% of the mAb. Human mAbs are the gold standard, and are generated from hybridomas of human or humanised mouse origin^[4]^. mAbs with the strongest affinities/biological response are selected using phage display systems^[5]^, or high throughput immunoassays^[6-8]^.

**Table 1:** Adapted list of monoclonal antibodies approved by FDA for cancer treatment

Active ingredient	Drug’s name (year of FDA approval)	Indications	Structure	Company	Mechanism of action	Important adverse events
Alemtuzumab	Campath (2001)	CLL (chronic lymphocytic leukemia)	Humanized IgG1 kappa	Genzyme Corporation	CD 52 binding, which leads to antibody-dependent lysis of leukemic cells	Infusion-related events (bronchospasm, rash, hypotension), immune-mediated diseases
Bevacizumab	Avastin (2004)	As part of combination therapy for metastatic colorectal cancer and HER-2 negative metastatic breast cancer	Humanized IgG1	Genentech Inc.	Decrease blood vessel proliferation by binding to VEGF (prevent interaction of VEGF with its receptors Flt-1, KDR)	Bleeding, rash, gastrointestinal perforation, allergic reactions, increased risk of infections
Cetuximab	Erbitux (2004)	EGFR-expressing metastatic colorectal carcinoma	Chimeric IgG1	Bristol-Myers Squibb (USA), Merck (EU)	Inhibit cell growth, induct apoptosis, reduce production of VEGF, by binding to epidermal growth factor receptors	Acne-like rash, photosensitivity, hypomagnesemia, infusion-related reactions
Gemtuzumab ozogamicin	Mylotarg (2017)	CD-33 positive acute myeloid leukemia (AML)	Humanized IgG4	Wyeth Pharms Inc.	CD-33 directed antibody-drug conjugate	Hepatotoxicity, haemorrhage, embryo-fetal toxicity
Ipilimumab	Yervoy (2011)	Unresectable or metastatic melanoma	Humanized IgG1	Bristol-Myers Squibb	CTLA-4 (Cytotoxic T-lymphocyte antigen-4) blocking antibody	Immune-related adverse events
Ofatumumab	Arzerra (2009)	CLL (chronic lymphocytic leukemia)	Human IgG1	Novartis	Antibody to CD20 protein	Respiratory infections, anaemia, neutropenia, rash
Panitumumab	Vectibix (2006)	Metastatic colorectal cancer	Human IgG2	Amgen	EGFR binding antibody	Skin rash, fatigue, nausea, diarrhoea, fever, hypomagnesemia
Pembrolizumab	Keytruda (2014)	Melanoma, non-small cell lung cancer, head and neck squamous cell carcinoma	Humanized IgG4 kappa	Merck	PD-1 (programmed cell death-1) blocking antibody	Immune-related adverse events
Rituximab	Rituxan (1997)	CLL (chronic lymphocytic leukemia), CD20-positive non-Hodgkin’s lymphoma	Chimeric IgG1	Genentech	Cell lysis, by binding to CD20 antigen on B lymphocytes	Skin rash, low blood pressure, hair loss, fatigue, cytokine release syndrome
Trastuzumab	Herceptin (1998)	HER2-positive breast cancer	Humanized IgG1	Genentech	HER2 (c-erb82) binding antibody	Nausea, diarrhoea, cardiac dysfunction (congestive heart failure, cardiomyopathy)
Avelumab	Bavencio (2017)	Metastatic Merkel cell carcinoma (MCC)	Human IgG1 lambda	AMD Serono	PD-L1 (programmed death ligand-1) blocking antibody	Immune-mediated diseases
Durvalumab	Imfinzi (2017)	Locally advanced or metastatic urothelial carcinoma	Human IgG1 kappa	AstraZeneca	PD-L1 (programmed death ligand-1) blocking antibody	Immune-mediated diseases
Brentuximab vedotin	Adcetris (2011)	Hodgkin lymphoma, systemic anaplastic large cell lymphoma (ALCL)	Chimeric IgG1	Seattle Genetics	CD30 antibody with MMAE (monomethyl auristatin E), which disrupts microtubule network in the cell	Chemotherapy-induced peripheral neuropathy, neutropenia, fatigue, nausea, anaemia, fever

Monoclonal antibodies have been developed to target cancer cells using a number of distinct and fascinating mechanisms. Naked antibodies that lack any type of drug conjugation work by either: (1) stimulating the immune system by binding to an antigen present on a cancer cell (alemtuzumab); (2) boosting the immune response via interaction with immune-checkpoint proteins (CTLA-4 inhibitors (ipilimumab)/PD-1 inhibitors (pembrolizumab); or (3) blocking growth factor receptors on cancer cells (trastuzumab). In contrast, conjugated (tagged, labelled, loaded) mAbs work by carrying radioactive elements [radiolabelled antibodies - ibritumomab tiuxetan (Zevalin)] or chemotherapeutic drugs [chemolabeled antibodies - brentuximab vedotin (Adcetris)]. An additional group of mAbs, called bispecific mAbs, possess two different antigen binding fragments (Fabs) whose function is to bring cells in proximity to one another. For example, blinatumomab binds CD19 on lymphoma cells and CD3 on T cells, thus prompting T cell cytotoxicity against leukemic B cells^[9]^.

### Adverse events and monoclonal antibody treatment

While mAbs are a promising new therapy for the treatment of a growing number of cancers, they can cause various systemic and cutaneous adverse events, including a wide range of hypersensitivities: antibody mediated type I reactions (anaphylaxis), cytotoxic type II (neutropenia, thrombocytopenia, haemolytic anaemia), immune complex type III (vasculitis), T cell mediated type IV (delayed mucocutaneous reactions, cardiac events, progressive multifocal encephalopathy (PML)^[10]^. Type I hypersensitivities are most common, with a recent study showing that among 901 patients treated with rituximab, 9% (*n* = 79) faced type I hypersensitivity reactions. The absence of IgE against rituximab, however suggested that the patients developed pseudo-allergic reactions, which manifest with the same clinical symptoms as true type I hypersensitivities (flushing, hypotension, mucous secretion, rash, rhinitis, conjunctivitis) but less severe^[11,12]^. Cytotoxic (type II) reactions manifest as neutropenia, anaemia, thrombocytopenia, and are common for rituximab and trastuzumab. This is perhaps due to their antitumor mechanism of action, which occurs via antibody- and complement-dependent cell cytotoxicity^[13]^. Type III reactions occur due to the formation of antibody-antigen complexes and are relatively rare in response to mAb treatment, with the exception of chimeric antibodies such as rituximab, where serum sickness-like reaction occur in up to 20% of individuals^[14]^. T-cell mediated type IV hypersensitivity reactions occur following cessation of mAb treatment from 12 h to several weeks. They vary from maculopapular rash to severe adverse events (Steven Johnson Syndrome, erythema multiforme), and are thought to be primarily T-cell mediated reactions, which explains the delayed onset of such toxicities^[15]^.

Prediction of immune-related adverse events and their prevention are essential milestones for the development of personalised cancer treatment. Pharmacogenetic studies will likely be crucial in the near future to understand individual adverse responses to mAb treatment and how they can be avoided. In spite of the fact that this problem is very topical for modern medicine, there are currently no published studies that have examined the association between individual genetic variations and the development of adverse reactions to mAb treatment.

### Therapeutic significance of pharmacogenetic study

Pharmacogenetics is a multidisciplinary research area that aims to predict and personalise modern treatment protocols by understanding the influence of genetic variation on drug efficacy and toxicity. Millions of genetic polymorphisms have thus far been identified in the human genome, many of which can affect pharmacokinetics and pharmacodynamics of antineoplastic drugs^[16,17]^. Understanding these genetic polymorphisms is particularly important in cancer research, as cancer cells possess an increased number of genetic mutations, some of which may influence drug transport, metabolism, toxicity and cellular response^[18]^. For non-mAb cancer treatments, the majority of these polymorphisms lie within genes responsible for drug transport and metabolism, and likely underlie inter-individual differences in drug response^[19]^. Genetic polymorphisms in genes, encoding enzymes TPMT (thiopurine methyltransferase), CDA (cytidine deaminase), and CYP2D6 (cytochrome P450 2D6) can lead to severe changes in the metabolism of non-mAb treatments such as mercaptopurine, azathioprine and tamoxifen respectively, but differ significantly from gene polymorphisms responsible for mAb metabolism and response^[20]^. Due to the immunological nature of mAbs, their efficacy can be affected by variations in genes responsible for antibody recognition, presentation and metabolism. This review will summarise the existing data concerning the influence of genetic variations on cancer treatment with mAbs.

## Part 1. The influence of genetic polymorphisms on the metabolism of monoclonal antibodies

**Figure 1:**
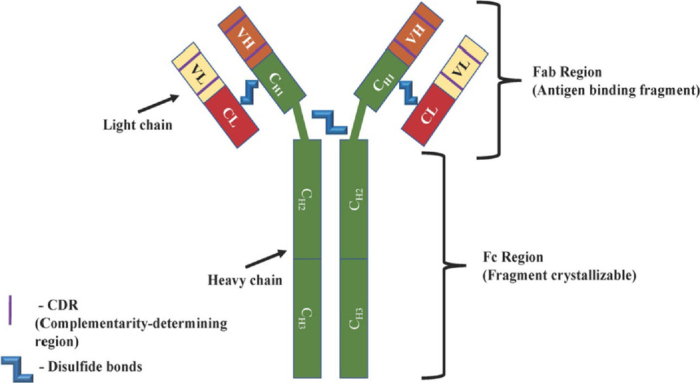
Structure of a monoclonal antibody

All current clinically available mAbs are IgG (immunoglobulin G) proteins, consisting of two heavy chains (50 kDa) and two light chains (25 kDa) composed of constant domains (C_H_ and C_L_) and variable domains (V_H_ and V_L_)^[21]^ [Figure 1]. The variable regions and C_H1_ domain comprise the Fab, which is specific for the target antigen. Together, C_H2_ and C_H3_ comprise the fragment crystallizable region (Fc), which can bind to cell surface receptors present on immune cell populations^[22]^. These membrane proteins known as Fc receptors are expressed on B lymphocytes, natural killer cells, macrophages and play role in the recognition of foreign antigens and neoplasms, as well as the activation of phagocytic and cytotoxic cells^[23]^. The half-life of mAbs is dependent on their structure: murine IgG have the shortest half-life of 1-2 days^[21]^. Chimeric IgG half-life is equal to 8-10 days and humanized or fully human half-life is 20-30 days^[24,25]^. This is significantly longer than traditional chemotherapies that possess half-lives ranging from hours (methotrexate) to 1-2 days (doxorubicin)^[26,27]^.

mAbs can be administered via intravenous (IV) infusion or subcutaneous (SC) and intramuscular (IM) injections. Due to the higher risk of infusion-related reactions following IV administration of mAbs, SC and IM are more preferable. Systemic absorption of mAbs from the injection site is slow, and a maximum concentration is usually reached in 1-8 days after SC or IM injection^[28]^. mAbs are distributed from the blood to tissues via convection, which is determined by the blood-tissue hydrostatic gradient, as well as by the sieving effect of the vascular epithelium^[29]^. Absorption of SC and IM administered mAbs is also dependent on factors such as gender, age, weight, blood pressure, respiratory rate and disease stage, and of course, genetic background^[30]^.

Metabolism of mAbs does not involve the cytochrome P450 enzyme system that is critically important for the hepatic metabolism of many cancer drugs^[31]^. Instead, due to their large molecular size, elimination of mAbs occurs mostly via endocytosis and pinocytosis followed by proteolytic catabolism^[32,33]^. Moreover, clearance can occur specifically (Fab or Fc receptor binding) or non-specifically^[34]^. Importantly, target mediated clearance is triggered by the interaction of the mAb with its antigen, and can therefore depend on specific tumour characteristics including the amount of antigen expressed. More frequently uptake of mAbs occurs via receptor-mediated endocytosis in response to binding of the antibody Fc domain to FcγR expressed on immune cells, such as monocytes, macrophages, dendritic cells^[35]^. This effect was emphasised in a 2008 study examining a polymorphism in the *FcgR3A* gene (rs396991 T/G) that results in a change of amino acid phenylalanine (F) to valine (V) at position 158. The valine substitution was shown to increase binding affinity and improve antibody-dependant cell-mediated cytotoxicity^[36]^. As a result, human epidermal growth factor receptor (HER) 2-positive breast cancer patients with the V/V genotype have higher response rate to treatment with anti-HER2 drug trastuzumab^[36]^. This mutation also increases the response rate and progression-free survival (PFS) of patients with colorectal cancer and B-cell lymphoma treated by cetuximab and rituximab, respectively^[36-38]^.

The expression of another Fc receptor termed the neonatal Fc receptor FcRn is also subject to genetic influence based on a variable number of tandem repeats (VNTR) in the promoter region of *FCGRT*, the FcRn gene. An increased number of tandem repeats has been shown to increase its expression^[39]^, and has subsequently been linked to increased serum infliximab and adalimumab in patients with inflammatory bowel disease^[40]^. As FcRn is responsible for salvaging IgG, reduced expression is thought to result in lower serum concentration and increased clearance via alternative mechanisms^[21]^. This polymorphism was also examined in patients treated with the anticancer epidermal growth factor receptor (EGFR) mAb cetuximab, where VNTR3 homozygotes possessing three repeats demonstrated a reduced distribution, clearance and a trend towards increased half-life (*P* = 0.058) of the mAb when compared to heterozygotes^[41]^.

## Part 2. The influence of genetic polymorphisms on monoclonal antibody treatments

### Association of growth signalling pathways with monoclonal antibodies response

**Figure 2:**
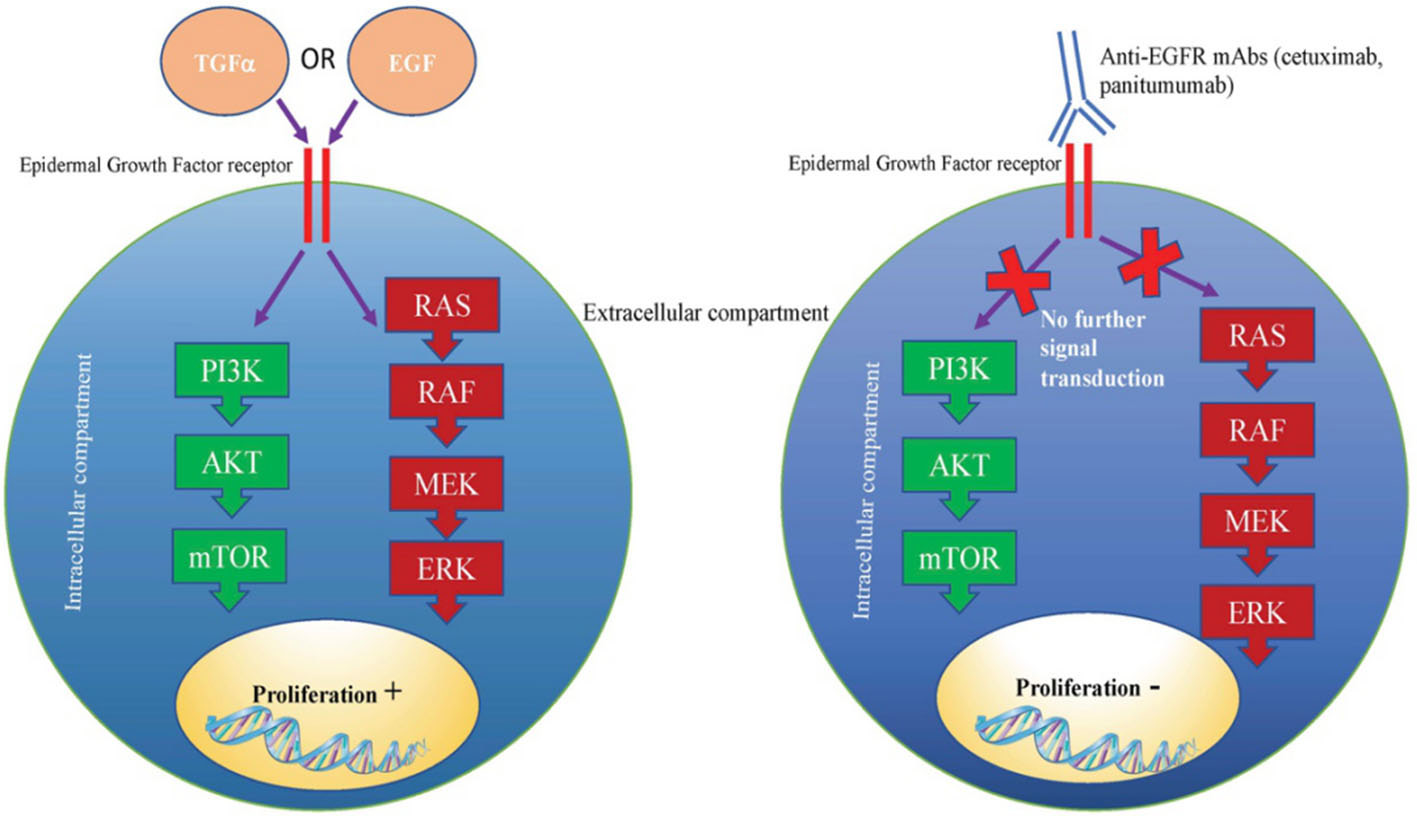
Mechanism of anticancer activity of anti-EGFR mAbs. Cetuximab and panitumumab bind to EGF receptors, thus preventing further signalling transduction via PI3K/mTOR and RAS/ERK pathways. Inactivation of growth signalling pathways prevents cell proliferation and survival. EGFR: epidermal growth factor receptor; mAbs: monoclonal antibodies

Mutations within growth signalling pathway genes are essential to fuel cancer development and progression. One such pathway that is targeted by mAb therapy is the EGFR pathway. Following EGF or Transforming growth factor alpha (TGFα) binding, EGFR signalling drives metastasis, proliferation and angiogenesis through activation of the Ras/Raf/ERK and PI3K/AKT/mTOR signalling pathways^[42]^. Anti-EGFR mAbs, such as cetuximab and panitumumab prevent receptor-ligand interactions, thus inhibiting downstream activation of growth signalling pathways [Figure 2].

Current pharmacogenetic research approaches have aimed to examine the potential relationship between somatic mutations in the Ras/Raf/ERK and PI3K/AKT/mTOR pathways, with efficacy of anti-EGFR mAbs used for treatment of colorectal cancer (cetuximab, panitumumab). Genetic polymorphisms of the gene encoding Raf protein (*BRAF*) are very crucial for cancer treatment with anti-EGFR mAbs, with *BRAF* mutations occurring in 4%-11% of patients with colorectal cancer^[43]^. In particular, *BRAFV600E* (rs113488022) is associated with an aggressive tumour phenotype, early lymph node metastasis and reduced response to cetuximab and panitumumab treatment^[44]^. A meta-analysis of 10 clinical trials has shown that cetuximab and panitumumab treatment did not improve PFS, overall survival (OS) and response rate in 462 colorectal cancer patients with *BRAFV600E* mutation (rs113488022) when compared to the control group treated by standard chemotherapy^[44]^. In the same study, Pietrantonio *et al.*^[44]^ have shown that patients without the *BRAFV600E* mutation were associated with lower risk of progression (*P* = 0.001) and higher response to cetuximab and panitumumab treatment (*P* = 0.001), compared to those with mutation. Interestingly, the *BRAFV600E* mutation has been shown to destabilise the inactive conformation of the Raf protein, rendering the new mutated protein in a constitutively active state^[45]^. Consequently, upstream inactivation of EGFR signalling via mAb treatment would have minimal therapeutic effect, explaining the clinical findings.

Several studies have examined the correlation between genetic polymorphisms affecting PI3K/mTOR signalling pathway and anti-EGFR mAb treatment. This pathway plays a crucial role in regulating the cell cycle. Frequent polymorphisms rs17849079 and rs7640662 within the *PIK3CA* gene (encoding p110α, the main subunit of PI3K) occur in 10%-18% of patients with colorectal cancer and are known as “hotspot mutations”^[46]^. A recent meta-analysis confirmed that rs17849079 allele T and rs7640662 allele C predict poor objective response rate, lower OS and PFS in patients with metastatic colorectal cancer, treated by cetuximab and panitumumab^[47]^. The *PTEN* gene, encoding phosphatase and tensin homolog (PTEN) protein is an inhibitor of the PI3K/mTOR pathway, and is strongly linked to a number of cancers. Several studies have demonstrated that *PTEN* single nucleotide polymorphisms (SNPs) rs701848 allele C, rs2735343 allele G and rs11202586 allele T are correlated with increased risk of oesophageal squamous cell cancer and testicular germ cell tumours^[48]^, however they have demonstrated no association with response to mAb treatment to date.

**Figure 3:**
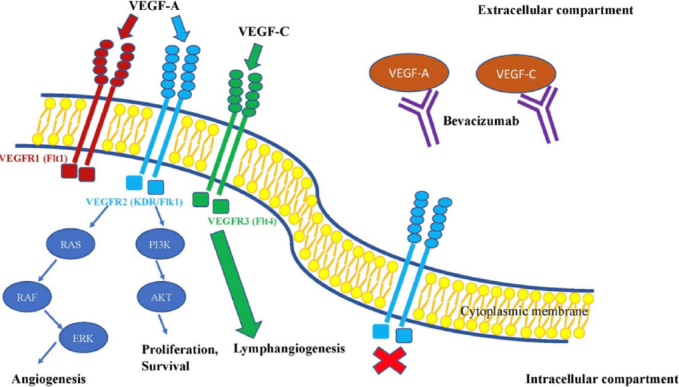
The effect of bevacizumab on VEGF signalling pathway. This figure shows, that bevacizumab is primarily directed to bind VEGF signalling molecules. Such interaction leads to inactivity of VEGF binding to its receptors and as a result it reduces neoangiogenesis. VEGF: vascular endothelial growth factor

Like other tissues, tumours require adequate vascularisation to proliferate. Vascular endothelial growth factor (VEGF) is a key signalling molecule in this process. The VEGF family consists of 5 members: VEGF-A, placenta growth factor, VEGF-B, VEGF-C and VEGF-D, all of which signal through unique receptors (VEGF receptors). There are three VEGF receptors, VEGFR1, VEGFR2 and VEGFR3, all of which possess unique roles and tissue distribution. Due to its role in angiogenesis however, the majority of cancer research has focused on VEGFR2 and to a lesser extent VEGFR3 for its role in lymphangiogenesis, a crucial driver of cancer metastasis^[49]^. Following VEGFR2 binding, Ras-ERK and PI3K-AKT pathways are activated to stimulate angiogenesis, proliferation and survival^[50]^. VEGF-A and VEGF-C are the key oncogenic drivers within the VEGF family, stimulating angiogenesis and lymphangiogenesis respectively^[51]^. Due to their angiogenic role in tumour formation, the VEGF pathway has become a therapeutic target, as exemplified by anti-VEGF mAbs such as bevacizumab and chemotherapeutic drugs Sunitinib and Sorafenib^[52]^ [Figure 3].

As with *EGFR*, *VEGF* genetic polymorphisms can influence individual response to anti-VEGF mAb therapies. In 2008 Schneider and colleagues demonstrated that *VEGFA* polymorphisms were associated with median OS for the treatment of metastatic breast cancer with bevacizumab^[53]^. In particular, patients with the *VEGF* rs699947 AA genotype, who were treated by combination of anti-VEGF bevacizumab and paclitaxel chemotherapy had better treatment response and improved OS, as compared to patients treated with paclitaxel only^[53]^. In the case of metastatic colorectal cancer, patients with the rs833061 TT *VEGF* genotype treated with bevacizumab in combination with folinic acid, fluorouracil and irinotecan showed a reduced risk of PFS^[54]^. In addition, SNPs rs699947 C>A and rs833061 C>T have both been shown to increase the expression of VEGF protein, and may account for the genotype based differences in anti-VEGF mAb response^[55,56]^.

Mutations in the *RAS* gene family also affect the response to bevacizumab treatment, where they are present in 30% of all human cancers^[57]^. In 2016, Fiala *et al*.^[58]^ reported that among 404 Caucasian patients with metastatic colorectal cancer, those with the *RAS* gene *KRAS G12A/V* (rs121913529) had lower PFS and OS comparing to patients with *KRAS* wild-type when treated with bevacizumab. In a group of bevacizumab treated Asian patients with *KRAS G12A/V* (rs121913529) polymorphism, objective response rate was also lower comparing to patients with wild type tumours^[59]^. Another study showed that among 167 patients who underwent resection of lung metastases for metastatic colorectal cancer, perioperative bevacizumab was correlated with better recurrence and OS in those who had *KRAS* exon 2 codon 12 mutations^[60]^. The beneficial effect of bevacizumab in patients with a *KRAS* codon 12 mutation was speculated to be due to its association with VEGF upregulation thus promoting angiogenesis^[61]^. As an inhibitor of VEGF signalling, bevacizumab may inhibit cancers that are reliant on VEGF mediated tumorigenesis^[62]^. The findings relating to treatment outcome eventually translated into clinical practice, with *KRAS* genotyping becoming an important determinant of EGFR mAb therapy usage^[63]^.

### Influence of genetic polymorphisms on the efficacy of immune-checkpoint inhibitors

A new class of highly effective mAbs known as immune-checkpoint inhibitors have recently come into use that target checkpoint proteins present on T cells. These cell receptors termed CTLA-4 and PD-1 are regulatory proteins that prevent excessive T cell responses, but that may become over-expressed in some cancers^[64]^, along with their respective ligands. Cytotoxic T-lymphocyte antigen-4 (CTLA-4) binds CD80 and CD86 on antigen-presenting cells^[65]^ thus downregulating the neoplastic immune responses and facilitating cancer growth^[66]^. Programmed cell death-1 (PD-1) binds PD-L1 and PD-L2 that are often highly expressed on cancer cells^[66]^. Novel immunotherapies are directed towards blocking CTLA-4 and PD-1, as well as its ligands PD-L1 and PD-L2. These mAbs are highly effective and specific, however they can cause a wide range of adverse events due to a subsequently unregulated T cell immune response.

SNPs within the PD-L1 gene *CD274* have been shown to influence patient response to the anti-PD-1 mAb nivolumab [Table 2]. Patients with non-small cell lung cancer treated by nivolumab possessing the *CD274* rs4143815 C/C and C/G genotypes had modestly higher median PFS when compared to patients with the G/G genotype (*P* = 0.044)^[67]^. In addition, individuals with the *CD274* rs2282055, G/G and G/T genotypes had a modest increase in median PFS compared to T/T carriers [2.6 months *vs.* 1.8 months (*P* = 0.0163)]^[67]^. It has been also suggested in several studies that PD-L1 rs4143815, which is located in the 3’ untranslated region (UTR) can influence the expression of PD-L1, thus driving tumour cell immune escape^[68-70]^. In particular, the C allele of rs4143815 has been shown to increase production of PD-L1 by attenuating miR-570^[71]^. Consequently, patients with the rs4143815 C/C genotype have an inferior clinical response to paclitaxel-cisplatin chemotherapy^[72]^. While these results contradict some of the work by Nomizo *et al.*^[67]^, it remains undetermined how rs4143815 in particular affects anti-PD-1 therapy. This study was limited by several factors including the small sample size and absence of PD-L1 expression data from tumour cells, and thus, requires further study.

**Table 2:** Summary of SNPs’ influence on cancer treatment with mAbs

SNP	Gene	Examined drugs	Type of cancer	Consequences
rs699947 (AA)	*VEGF*	Bevacizumab (in combination with paclitaxel)	Metastatic EGFR-2 negative breast cancer	Higher treatment response and OS
rs833061 (TT)	*VEGF*	Bevacizumab (in combination with folinic acid, fluorouracil and irinotecan)	Metastatic colorectal cancer	Reduced PFS
rs121913529 (*KRAS G12A/V*)	*KRAS*	Bevacizumab	Metastatic colorectal cancer	Reduced PFS and OS
rs113488022 (*BRAFV600E*)	*BRAF*	Cetuximab, Panitumumab	Colorectal cancer	Early lymph node metastasis, lower response
rs4143815 (CC)	*PD-L1*	Nivolumab	Non-small cell lung cancer	Higher PFS
rs4553808 (G) rs11571317 (T) rs231775 (A)	*CTLA-4*	Ipilimumab	Metastatic melanoma	Higher response, associated with grade 3-4 immune-related adverse events
rs2282055 (GG)	*PD-L1*	Nivolumab	Non-small cell lung cancer	Higher median of PFS
rs396991 (G)	*FcgR3A*	Trastuzumab	HER-2 positive breast cancer	Higher response rate
		Cetuximab	Colorectal cancer, B-cell lymphoma	Increase response rate and PFS
		Rituximab		
rs733618 (G)	*CTLA-4*	Ipilimumab, Tremelimumab	Metastatic melanoma	Higher response rate
rs4553808 (G)	*CTLA-4*	Ipilimumab	Metastatic melanoma	Higher risk of endocrine immune-related adverse events
rs733618 (G) rs3087243 (G)	*CTLA-4*	Ipilimumab	Metastatic melanoma	Higher long-term survival at 3-4 years, comparing to heterozygous
rs17849079 (T) rs7640662 (C)	*PIK3CA*	Cetuximab, Panitumumab	Metastatic colorectal cancer	Poor objective response rate, lower OS and PFS

SNPs: single nucleotide polymorphisms; PFS: progression-free survival; OS: overall survival

*CTLA4* gene polymorphisms are also associated with the response to anti-CTLA-4 treatment. In 2008, Breunis *et al.*^[73]^ reported that alleles G of rs4553808, T of rs11571317 and A of rs231775 were significantly associated with improved response to CTLA-4 blockade treatment but increased severity (grade III/IV immune-related adverse events) in patients with metastatic melanoma. Moreover, during the haplotype analysis, which included seven SNPs (rs733618, rs4553808, rs11571317, rs5742909, rs231775, rs3087243 and rs7565213), it was suggested that haplotype TACCGGG could be correlated with no response and TGCCAGG with a response to anti-CTLA-4 treatment. However, no statistical significance was detected^[73]^. In 2013, Queirolo *et al*.^[74]^ examined 6 *CTLA-4* SNPs: rs231775 (+49A>G, exon 1), rs4553808 (-1661A>G), rs5742909 (-319C>T, promoter), rs11571316 (-1577G/A), rs11571317 (-658C>T, 5’UTR) and rs3087243 (CT60G>A, 3’UTR) in 14 patients with metastatic melanoma treated with ipilimumab or tremelimumab. It was detected that 83% (5/6) of responders possessed the rs733618 G genetic variant of *CTLA-4* gene^[74]^. It was also reported, that all responders had diplotype GG - AA (-1577G/CT60G and -1577A/CT60A). In addition, the rs3087243 heterozygous of *CTLA-4* gene was correlated with better 5-year survival, compared to patients with homozygous genotype (*P* < 0.001). In this study, all other CTLA-4 SNPs were not statistically correlated with response to the treatment or OS^[74]^. In a more recent study by Queirolo *et al.*^[75]^, patients with stage IV melanoma, treated by ipilimumab, possessing rs11571316 (-1577G/A) and rs3087243 (CT60G>A) homozygous genotypes had better long-term survival at 3 and 4 years, compared to heterozygous (G/A) genotypes. Because patients with the homozygous GG genotype possess reduced CTLA-4 mRNA and protein expression, it was suggested that the efficacy of ipilimumab was increased in these individuals^[76]^. In addition, a 2018 study by Queirolo *et al*.^[77]^, found that the rs4553808 (-1661G/G) was higher among patients with endocrine immune-related adverse events (irAEs) but not cutaneous or gastrointestinal adverse events.

### The future of monoclonal antibody therapies: personalised care?

Current data suggests that mAbs are effective and specific anticancer drugs, however their efficacy and toxicity have demonstrated significant variability due to genotypic differences relating to mAb recognition, metabolism and cancer signalling. Consequently, next-generation sequencing (NGS) technologies, and particularly whole-genome sequencing (WGS) and whole-exome sequencing (WES) will prove to be invaluable as mAbs become more widely used. While WGS sequences the genome in its entirety (> 95%), WES sequences only transcribed regions, and is therefore significantly faster and cheaper. Nonetheless, WGS captures significantly more information, as it can identify significant mutations that are not transcribed such as promoter regions. As the cost of NGS decreases, usage will increase, thus allowing the identification of the full range of somatic mutations present in cancerous and non-cancerous tissue, and allowing physicians to decide on the best therapeutic options^[78]^.

A prime example of NGS research into mAb therapies was performed by Rizvi *et al.*^[79]^, who performed WES on non-small cell lung cancers treated with pembrolizumab. Paradoxically, a higher number of nonsynonymous somatic mutations (*n* ≥ 302) resulted in improved efficacy of pembrolizumab treatment, as indicated by a more durable clinical benefit, higher objective response rate (ORS) and PFS (*P* = 0.02). It was demonstrated that an increase in the number of somatic mutations increased the production of T cell-reactive neoantigens. Because these tumour neoantigens are recognized as foreign, they stimulate a stronger T-cell immune response, particularly in combination with anti-PD-1 mAb treatment^[79]^.

## Conclusion

In summary, the influence of genetic background on cancer treatment is not limited to chemotherapies. mAb efficacy and metabolism can be significantly impacted by host genetics, whether it be non-synonymous mutations altering protein structure and function or promoter mutations affecting gene regulation. Host polymorphisms can also affect mAb target binding, thus significantly affecting treatment efficacy. Key mutations within critical signalling pathways can affect overall and progression free survival, as well as treatment response and treatment-related toxicity. Pharmacogenetics is an essential tool to recognise the association between such germline or somatic mutations and efficacy or toxicity of mAbs in cancer treatment. It provides the potential to personalize cancer therapy with mAbs and other chemotherapies with respect to drug choice, drug combination, and dosing. A better understanding of pharmacogenetics in cancer treatment will undoubtedly benefit existing treatment protocols by implementing new genetic screening methods such as NGS into clinical practice prior to treatment initiation. Such screening will allow physicians to predict drug pharmacokinetics and pharmacodynamics, as well as choose the most appropriate mAb treatment for individualised cancer management.

## Declarations

### Copyright

© The Author(s) 2019.
